# Pentoxifylline and tocopherol for the treatment of osteoradionecrosis of the jaws. A systematic review

**DOI:** 10.4317/medoral.25729

**Published:** 2023-01-15

**Authors:** Marcelo Arqueros-Lemus, Dylan Mariño-Recabarren, Sven Niklander, René Martínez-Flores, Víctor Moraga

**Affiliations:** 1 Oral and Maxillofacial Surgery, Dentistry Faculty, Universidad Andres Bello, Viña del Mar, Chile; 2 Oral Pathology and Oral Medicine Unit, Dentistry Faculty, Universidad Andres Bello, Viña del Mar, Chile; 3 Maxillofacial Surgery and Dentistry Service, Carlos Van Buren Hospital, Valparaíso, Chile

## Abstract

**Background:**

Osteoradionecrosis of the jaws (ORNJ) is a severe and challenging complication of head and neck radiation therapy. Despite its aggressiveness and controversy respect to its efficacy, surgical intervention remains the main treatment modality. Nevertheless, due to advances in the understanding of ORNJ physiopathology, new treatment alternatives such as the combination of pentoxifylline with tocopherol (PENTO) have emerged. The aim of this systematic review was to assess the reported efficacy of PENTO for the treatment of ORNJ.

**Material and Methods:**

Studies were search using Pubmed, The Cochrane Library, Scopus, and Web of Science data bases following the PRISMA guidelines. Inclusion criteria were cohort, case series, randomized or non-randomized clinical studies published in English including human subjects who received PENTO as treatment for ORN of the jaws.

**Results:**

Eleven articles met the inclusion criteria and were included for data analysis. All studies reported patients with complete mucosal coverage with no exposed bone (considered healthy) after PENTO treatment, ranging from 16.6% to 100% of the patients, depending on the study. Clinical improvement or disease stabilization was reported between 7.6% and 66.6% of studied individuals, while disease progression was seen in only 5 studies involving 7.6 - 32% of patients.

**Conclusions:**

PENTO treatment achieved a complete disease control in a significant number of patients in all studies. However, there is no standardized protocol for administering the therapy. It is necessary to determine the pharmacological doses and to evaluate the benefits of adding antibiotics and clodronate. Good quality clinical trials are needed to develop a successful algorithm for the management of ORN of the jaws.

** Key words:**Osteoradionecrosis of the jaws, radiotherapy, pentoxifylline, tocopherol, vitamin E.

## Introduction

Osteoradionecrosis of the jaws (ORNJ) is defined as an area of devitalized, exposed bone due to head and neck radiation therapy, which is unable to heal after a period of 3 to 6 months without any local signs of neoplastic disease (1). It is considered a severe, difficult to manage complication of head and neck (HN) radiotherapy. The prevalence of ORNJ varies between different studies, but it has been recently estimated between 5-12% of all patients receiving HN radiotherapy (2). The reported annual incidence is of 4.8% (3), which increases to 7% if the patient had a tooth extracted (4). 

ORNJ has variable clinical presentations, including asymptomatic intraoral exposure of small areas of bone, to large intra and extraoral bone exposures, oral and cutaneous fistulas, and pathological fractures (5). Most common symptoms are trismus, sensorineural disturbances (anesthesia, dysesthesia, and paresthesia), speaking difficulties and pain during mastication (6,7). 

One of the first theories explaining the pathogenesis of ORNJ was proposed by Meyer in 1970. He suggested that ORNJ develops due to traumatic events in previously irradiated tissues, which predisposes to infective processes from the surrounding microflora. Because of that, the main treatment of choice for ORNJ at that time were antibiotics. Nevertheless, clinical results were not good (8). Years later in 1983, Marx proposed the “3H” hypothesis (hypoxia, hypovascularity and hypocellularity) to explain the development of ORN (1). This hypothesis suggested that radiation therapy induces homeostatic and metabolical changes in adjacent tissues resulting in reduced vasculature and high oxygen demand, which lead to an hypoxic, hypocellular and hypovascular bone prone to necrosis (1). Lately, Delanian and Lefaix proposed a theory called radiation-induced fibro-atrophic process (RIF), which suggests a progressive destruction of the bone matrix due to an indirect effect of reactive oxygen species (ROS) and to direct damage to endothelial cells and fibroblasts, leading to microvascular necrosis and an alteration in collagen metabolism (9). Up to date, this is the most accepted hypothesis to explain ORNJ pathogenesis. 

Different treatment modalities for ORNJ have been reported, being conservative and surgical therapies considered as conventional therapies. Conservative treatment includes curettage, gentle elimination of bone sequestration and regularization of bone irregularities of exposed bone to prevent the development of new lesions (10). If this approach fails, surgical treatments need to be considered. The most common surgical treatment is deep surgical debridement through bone curettage (5,11). Other surgical options include sequestrectomy, fistula closure and mandibular resection with microvascular graft reconstruction (12). Despite there is an ongoing debate about the effectivity of surgical therapies, they still represent the main treatment of choice and the one that shows better results (13). 

Based on the RIF theory, Delanian in 2002 reported a new pharmacological approach based on the combination of tocopherol (VitE) and pentoxifylline (PTX) for the management of ORNJ (14,15). Since then, different studies have shown that the combination of PTX with tocopherol (PENTO) has promising results for the treatment of ORNJ (12,14,16), and despite the lack of robust evidence regarding its efficacy, this treatment regimen is currently used by some clinicians (17). The aim of this study was to perform a systematic review to assess the reported efficacy of PENTO for the treatment of ORNJ. 

## Material and Methods

This systematic review was performed following the recommendations from PRISMA for systematic reviews (18), to answer the following question: Is the combined therapy of pentoxifylline and tocopherol (PENTO) effective in the treatment of osteoradionecrosis of the jaws?

- Eligibility criteria

Inclusion criteria: articles in English, studies reporting the use of PENTO as treatment agent for ORN affecting the maxilla, mandible, or both. Only cohort, case series (with n > 10) and randomized or non-randomized clinical studies were included.

Exclusion criteria: animal or *in vitro* studies, studies reporting the use of PENTO in conditions different to ORNJ, such as osteomyelitis and medication induced osteonecrosis of the jaws.  

- Information sources and search Strategy 

A literature search was performed using four databases: PubMed, Scopus, Web of Science and the Cochrane Library. The search was performed between May 1 and June 2021. We used the following keywords: "osteoradionecrosis", "pentoxifylline", "tocopherol", "vitamin E" and “jaws”, which were combined with the Boolean operators “AND”, “OR” and/or "NOT".  We also performed a manual search complementary to the previous described strategy. 

- Selection process

All articles were independently reviewed by two reviewers (M.A.L and D.M.R). First, all duplicates were removed. Then, the remaining articles were selected by title and abstract. Next, full texts were revised and articles meeting the inclusion criteria were included for data extraction. All disagreements were discussed with a third reviewer (V.M.G), who had the final decision whether the study had to be included or not. 

- Data extraction

Two reviewers (M.A.L y D.M.R) extracted the following data: author; year; country; size; ORN-RT latency; PENTO protocol; other intervention used; lesion progression; sTable /improved; healthy; healing rate and Follow-up.

- Risk of bias assessment

Methodological quality assessment of the included cohort studies was carried out using the Newcastle-Ottawa System (NOS) (19). Two authors (M.A.L and D.M.R) independently assessed all included reports. In case of disagreement, the basis for study quality was determined after a joint discussion with a third reviewer (RMF). The three categories evaluated were, 1) selection of study groups, 2) comparability of study groups and 3) outcome. The quality instrument of the Newcastle-Ottawa scale is scored by awarding one point for each response marked with an asterisk on the scale. The possible scores are 4 points for selection, 2 points for comparability and 3 points for results, obtaining a maximum of 9 total points. Studies were divided into three categories: low risk of bias (≥ 7 stars), moderate risk of bias (5-6 stars), and high risk of bias (≤ 4 stars). 

For articles that were not analytical studies, we used the Joanna Briggs Institute (JBI) Critical Appraisal Checklist for Assessing the Methodological Quality of Nonrandomized Experimental Studies (20) and Case Series (21). An overall assessment of each article was made, determining whether the risk of bias is low, high, or unclear (more information needs to be sought). We considered low risk of bias if 'yes' answers were ≥50%, high risk of bias if 'no' answers were ≥50%, and unclear risk of bias if 'unclear' answers were ≥50%.

## Results

**Figure 1:**
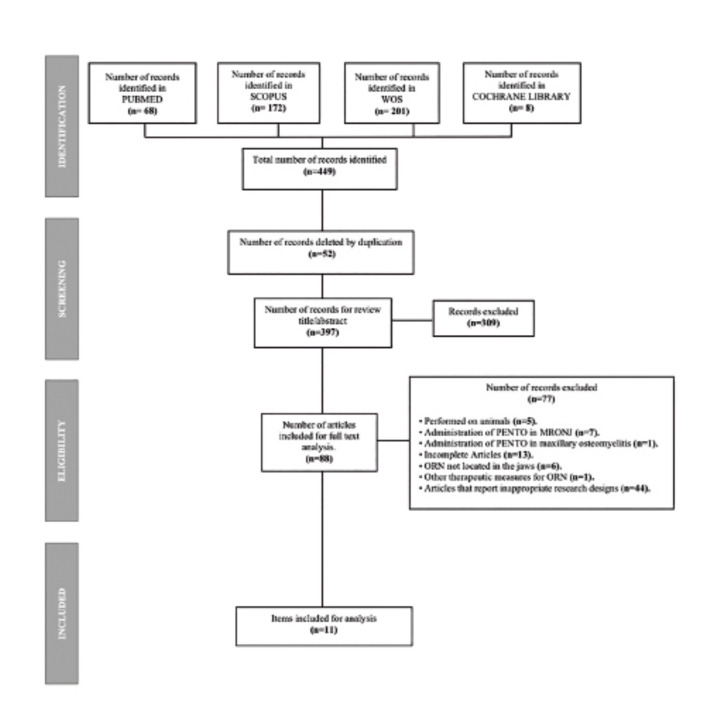
PRISMA flowchart.

We initially identified 449 articles for revision. Fifty-two articles were removed after duplicate removal, leaving 397 articles for screening by title and abstract. Of those, 309 were removed as were not related to the research question, leaving 88 papers for full text analysis. Only 11 articles met the inclusion criteria and were included for data extraction (Fig. 1). All included studies were published between the years 2005 and 2021 from four countries: France (12,14,16,22), United States (23) United Kingdom (5,7,11,24,25) and Brazil (26). In respect to study designs, 8 studies corresponded to cohort studies (5,7,11,16,22-25), 2 to non-controlled phase II clinical trials (12,14) and 1 to a cases series study (26). 

- Osteoradionecrosis of the jaws

The total irradiation dose was described only in 8 out of 11 articles and it ranged between 40 and 136 Gy (5,12,14,16,22,23,25,26). The elapsed time between RT and the development of ORNJ was mentioned in 8 articles and ranged between 1.7 and 5.3 years (5,12,14,16,22,24-26). The main trigger for the development of ORNJ was tooth extraction (12,14,23-26), followed by spontaneous appearance without an identified trigger and by periodontal and endodontic infections (23).

- PENTO therapy

Four articles mentioned the administration of antibiotics, corticosteroids and antifungals 2-4 weeks prior to the start of PENTO treatment (12,14,16,22). On the other hand, the use of antibiotics before administering PENTO therapy is mentioned in 2 articles (7,25). In 10 studies the PENTO regimen consisted of an intake of 400 mg of PTX twice a day with 1000 IU of tocopherol (5,7,11,12,14,16,22-25), while in one article the PENTO regimen consisted of 400 mg of PTX with 1200 mg of tocopherol daily (26). Together with the PENTO therapeutic scheme, antibiotics were administered in one article (5), but also in 3 studies due to secondary infection (7,11,25). Five studies supplemented this regimen with 1600 mg of clodronate (PENTOCLO) for the first 5 days of therapy (12,14,16,22,25). Two of these studies also added corticosteroids (16,22) and 2 added corticosteroids and antibiotics (12,14). The follow up period varied between 1 and 119 months and the average healing times ranged between 3.6 and 13.5 months (5,11,12,14,16,22,23,25,26). All studies reported some patients with full mucosal coverage without exposed bone (considered as healthy) after PENTO treatment, ranging between 16.6%- 100% of all patients, depending on the study (Table 1). 

Clinical improvement or disease stabilization was reported between 7.6% and 66.6% of studied individuals (5,7,12,14,22-26), while disease progression was observed only in 5 studies affecting 7.6-32% of the patients (Table 1).  PENTO therapy achieved a percentage of healthy patients that varied between 16.6% and 84.6% of the total number of individuals (5,7,11,23,24,26), while the addition of clodronate to the PENTO therapy obtained a success rate of 54.4 - 100% (12,14,16,22,25).  The mean healing time ranged from 3.6 to 13.5 months (5,11,12,14,16,22,23,25) (Table 1).

Four articles described mild adverse effects resulting from PENTO therapy: nausea (12,16), epigastric pain (12,16,26), diarrhea (16), headache (12), asthenia (12,16), insomnia (12,16) and palpitations (26). One of these articles did not specified and only mentioned “an adverse side effect of pentoxifylline” (24). One article described nausea as an adverse effect resulting from the use of clodronate (22), while in two articles they did not report adverse effects (14,23). In the remaining four articles, adverse effects were not mentioned (5,7,11,25).

- Risk of Bias Assessment

Of the 11 studies, 4 showed high risk of bias (7,16,23,24), 3 moderate risk (11,22,25) and 4 showed low risk of bias (5,12,14,26) (Table 2).

**Table 1:**
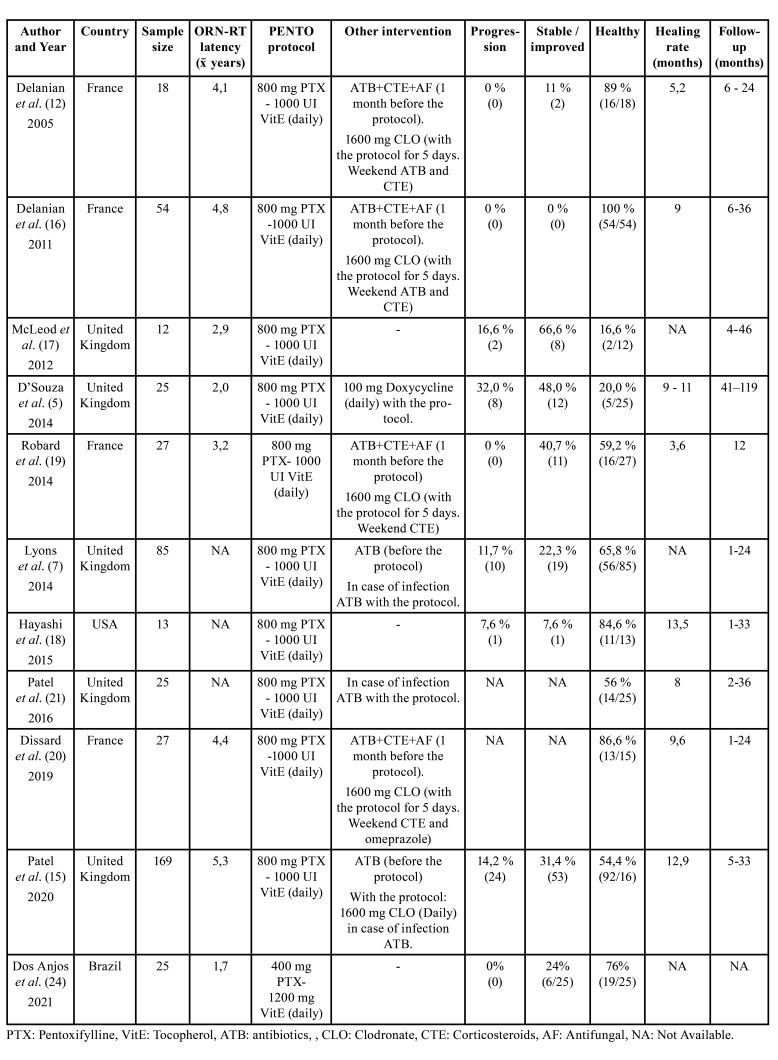
Summary of reviewed articles.

**Table 2:**
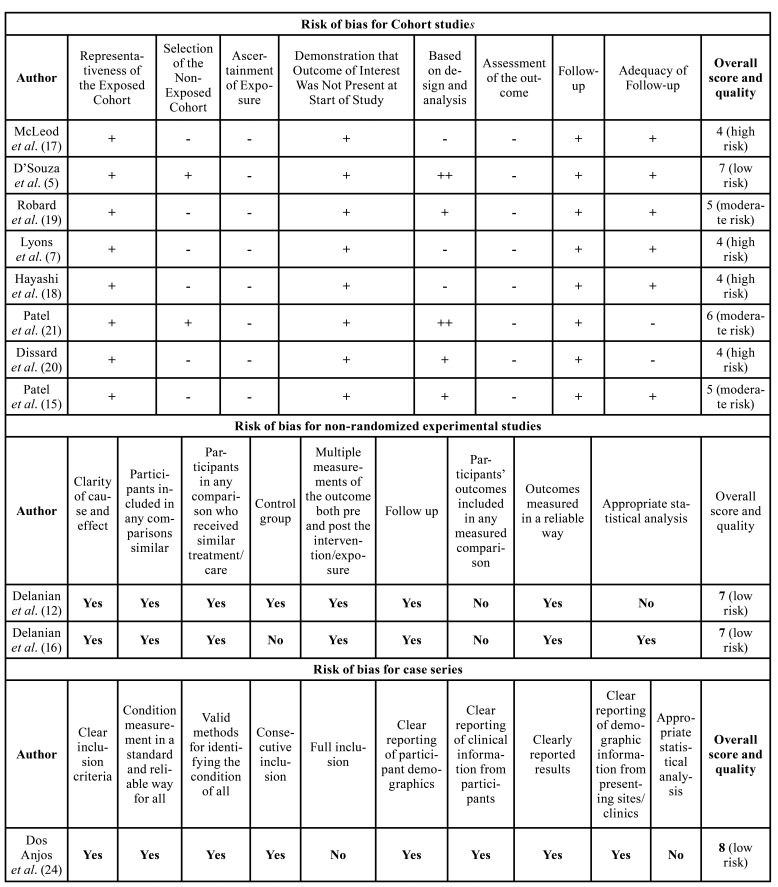
Risk of bias analysis for cohort, non-randomized experimental studies and case series.

According to the Newcastle-Ottawa System (NOS), the vast majority of studies did not present a description of how the selection of the cohort was carried out, so the selection item presented limited results (7,16,22,25). Three articles did not show comparability based on design and analysis between groups (7,23,24). Most of the studies adequately reported the results (5,7,16,22-25), however, one study presented flaws in this aspect (11). Both non-randomized experimental studies received a score of 7/9, considered as low risk of bias (12,14). The case series study received a score of 8/10, considered also as low risk of bias (26) (Table 2).

## Discussion

More than 100 years of research and inquiry have passed since the first reported case of ORN, and its pathophysiology is still a matter of debate. Up today, the most accepted theory is the radiation-induced fibro-atrophic process (RIF), and since its proposal, the use of pentoxifylline and tocopherol (PENTO) was introduced in the treatment of ORNJ (14). PTX is a methylxanthine derivative that exerts an anti-TNFα effect, increases vasodilation and erythrocyte flexibility, and reduces fibroblast proliferation, while increasing collagenase enzyme activity (27). On the other hand, tocopherol (Vitamin E) has an antioxidant action and inhibits the expression of procollagen genes, reducing fibrosis of the affected tissue (28). This way, these drugs act synergistically as potent antifibrotic agents (28,29).

The first clinical trial that assessed PENTO for the treatment of ORNJ was published in 2005. Eighteen patients with ORNJ were treated with PENTO, and in cases where no improvement was observed during the first 3 months, clodronate was added. In total, 16 patients (89%) achieved total mucosal coverage (14). Similar results have been also reported by others. Hayashi *et al*. (23) treated 13 ORNJ patients with PENTO and reported a complete visual resolution in 84.6% of the cases after an average of 13.5 months. However, no objective measures were employed to assess treatment response (other than visual inspection) and no details were given if the cases were in early or in advance stages of the disease (23), which seems to be of importance, as some authors have failed to obtain similar success rates when including ORNJ cases in advance stages. A retrospective study of 85 ORNJ patients treated with PENTO (and surgery when needed) reported a complete resolution in 65.8% of the cases, with a clear curative benefit of PENTO in mild and moderate cases, but more limited in advanced cases (7). Similarly, another study that included 10 advanced ORNJ cases between their cohort of 25 patients, reported a success rate of only 20% (*n*=25) (5). Considering these results, it seems that PENTO therapy can be beneficial, even curative, during early stages of ORNJ, but not in advance stages.

One study compared PENTO as a single therapy with other treatment alternatives, such as PENTO with antibiotics, PENTO with hyperbaric oxygen (HBO) and PENTO with surgical management. The highest treatment success was observed in the PENTO + surgery group (60%), followed by PENTO only group (56%) and PENTO + antibiotics group (27%). No resolution was observed in the PENTO + HBO group (11), which agrees with the results from Annane *et al*., who showed that HBO therapy had no advantage over any other treatment method (30). Another study compared the use of PENTO with HBO and local debridement (31). The success response of PENTO was not good, however, the number of patients who required mandibular resection and free flap reconstruction was lower compared to patients treated with HBO and debridement (5).

Various authors suggest the addition of clodronate in advance or refractory cases of ORNJ, showing high response rate (12,14,16,22,25). Clodronate is a first-generation non-nitrogenous oral bisphosphonate which reduces osteoclast activity and is used for the treatment of osteoporosis and hypercalcemia secondary to malignant neoplasms (32). Although bisphosphonates are clearly implicated in medication-associated osteonecrosis of the jaw (MRONJ), clodronate is the exception, because is the only bisphosphonate with the ability to stimulate osteoblasts, promote bone formation and decrease proliferation of osteoblasts and fibroblasts, enhancing the antifibrotic effect of PENTO (14,32).

A study that evaluated the use of PENTO associated with clodronate, and in certain cases complemented with sequestrectomy, reported a success rate of 86.6%. The authors concluded that the use of clodronate could avoid surgical debridement and increased clinical improvement (16). Delanian *et al*. evaluated the combination of PENTO with clodronate in ORNJ cases refractory to conservative treatment with HBO and surgery. In 100% of the cases (*n*=54), the lesions healed after an average of 9 months of treatment (12). McLeod *et al*. used the PENTO therapeutic protocol described by Delanian *et al*. (12) in 12 patients, but they did not administrate clodronate in severe cases of ORNJ. They reported healing in 16.6% of the patients but with many patients achieving disease stabilization (24). Dispensing clodronate in severe cases of ORNJ could explain the low success rate obtained in the study from McLeod *et al*. (24). Nevertheless, there is no robust evidence to support that statement, therefore, more RCTs are needed to confirm the efficacy of clodronate in severe cases of ORNJ.

Currently, there is no validated protocol that establishes the therapeutic doses of PENTO for ORNJ treatment and other complementary alternatives. Although most of the studies use a daily intake of 800mg of PTX with 1000 UI of Vitamin E, other therapeutic regimens have been used. Therefore, the heterogeneity in drug administration makes difficult to compare between different studies. It is also not clear how long the patient should remain under treatment. It has been pointed out that treatment should be administered for at least 6 months or when obtaining clinical resolution (14), but it should be considered that severe cases may require longer treatment periods (12,14). Well-designed clinical studies are needed to determine the ideal duration of PENTO therapy.

Some authors are in favor of administering preparatory interventions before starting PENTO therapy. Antibiotic and anti-inflammatory treatment for 2 to 4 weeks before starting with PENTO, to minimize the acute inflammatory phase and resolve any infectious condition, have been used by most of the studies (7,12,14,16,22,25). Interestingly, the highest percentages of healthy patients were reported in those reports. As for the studies where antibiotics were used simultaneously with PENTO, they did not report great treatment success (5,11). This could be explained by the fact that infection could affect the medications coverage of the affected area, causing treatment failure. PENTO therapy presented excellent adherence and tolerance by patients, reporting mild adverse-side effects, such as nausea, epigastric pain, diarrhea, headache, palpitations, asthenia, and insomnia in a minimum number of patients, which improved considerably with dose adjustment (24).

There are some limitations of the conclusions that can be drawn from this review. Most of the studies included here were retrospective, and some of them presented high risk of bias. In addition, there was great heterogeneity between different studies in terms of follow-up times, therapeutic protocols, classification systems and preparatory/complementary interventions, which makes the creation of a treatment algorithm very difficult.

## Conclusions

PENTO treatment is an effective treatment for mild to moderate cases of ORNJ, but not in advanced lesions. For advance stages of the disease, surgical intervention is still the first treatment option, nevertheless, before and after surgical treatment PENTO therapy is also advisable. The development of a treatment algorithm is desirable, but more standardized clinical trials are necessary to determine proper pharmacological doses of PENTO. Also, to reduce heterogeneity between the outcomes reported in clinical trials, the development of a core outcome set for assessing ORNJ is also needed.
